# Green and simple production of graphite intercalation compound used sodium bicarbonate as intercalation agent

**DOI:** 10.1186/s13065-022-00808-y

**Published:** 2022-03-15

**Authors:** Xin Wang, Guogang Wang, Long Zhang

**Affiliations:** 1 School of Petrochemical Technology, Jilin Institute of Chemical Technology, Jilin, 132022 China; 2 School of Information and Control Engineering, Jilin Institute of Chemical Technology, Jilin, 132022 China; 3 Jilin Provincial Engineering Laboratory for the Complex Utilization of Petro-resources and Biomass, School of Chemical Engineering, Changchun University of Technology, Changchun, 130012 Jilin People’s Republic of China

**Keywords:** Graphite intercalation compound, Green production, Mechanical force and chemical method

## Abstract

In view of the technical difficulties in the preparation of graphite intercalation compound (GIC) such as complex processes, the need to use strong acid reagents, and the product containing corrosive elements. A novel, efficient and simple method used sodium bicarbonate as intercalation agent was developed, which combined with mechanical force and chemical method for the green production of GIC. The production parameters were optimized by the single factor experiments, the optimal conditions were the ball mill speed 500 r/min for 4 h (6 mm diameter of the stainless-steel beads as ball milling media), the decomposition temperature 200 ℃ for 4 h, and 1:1 mass ratio of flake graphite to sodium bicarbonate. SEM results revealed that the prepared product appears the lamellar separation, pores, and semi-open morphology characteristic of GIC. FT-IR results indicated that the preparation method does not change the carbon-based structure, and the sodium bicarbonate intercalant has entered the interlayer of graphite flakes to form GIC. XRD results further showed that the GIC products still maintained the structure of carbon atoms or molecules, and the sodium bicarbonate intercalation agent has entered the interlayer of the graphite, and increased the interlayer distance of the layered graphite. The expandability of GIC products was studied, and the results show that it was expandable, and the expandable volume of GIC products prepared under optimal conditions has reached 142 mL/g. The theoretical basis for large-scale production was provided by studied the mechanism of the preparation method and designed the flow chart. The method has the advantages of simple process, products free of impurities, no use of aggressive reagents, process stable, and does not pollute the environment, being favorable to mass production, and provided new preparation method and idea for two-dimensional nanomaterials with preparation technical difficulties.

## Introduction

Carbon is abundantly distributed on the earth, and it can constitute many carbon materials with special properties. Graphite is an allotrope of carbon, it has excellent properties such as corrosion resistance, good heat resistance, and stable chemical properties [1]. The various excellent properties make it have broad application prospects in many fields [2]. In recent years, it was found that graphite intercalation compound (GIC) can be obtained by appropriate treatment of graphite. GIC maintains the planar hexagonal layered structure, and at the same time, the intercalation material interacts with the carbon layer, which changes some structural parameters between the layers and the layers. Therefore, GIC maintaining the excellent properties of graphite, such as high conductivity, light weight, and high specific surface area. At the same time, GIC also shows many special properties such as resistance to corrosion and oxidation, resistance to high and low temperatures, and so on [3]. Studies have shown that expandability is one of the important indicators of GIC products in practical applications. Expandable GIC can quickly decompose and generate a large amount of gas at suitable temperature, which makes graphite expanded dozens or even hundreds of times along the C axis, making it have important industrial value and industrial application prospects [4]. So far, the most popular methods for the preparation of GIC include chemical oxidation [5], electrochemical oxidation [6], vapor diffusion method [7] and ultrasonic oxidation [8]. The above preparation methods are often use aggressive reagents and restricted by the relatively high energy consumption, complex operation, environmental pollution, sometimes lower yield and poor product quality. Liquid phase method has been extensively studied because it is easy to operates and can obtain higher quality product [9]. However, the use of excessive organic solvents often leads to product instability, environmental pollution and increases production expense. Therefore, it is necessary to develop a novel greener production method to resolve the problems mentioned above.

To achieve these goals, we design a new method for the simple and green production of GIC from flake graphite. The effect of the production parameters (such as ball milling media, ball milling media size, ball milling time, ball mill speed, decomposition temperature, decomposition time and mass ratio of flake graphite to sodium bicarbonate) were investigated systematically. At the same time, the expandability of GIC under different production parameters was also studied. The morphology and structure of the obtained GIC samples were characterized and confirmed by SEM, XRD, and FT-IR, and the reaction mechanism was obtained. The process flow was also designed. This work has academic and industrial reference value for the preparation of GIC.

## Experimental

### Materials and instruments

The flake graphite (0.5 mm) and the sodium bicarbonate (AR) were purchased from Sinopharm Chemical reagent Co. (Shanghai, China).

Ball mills (QM-3SP04, YXQM-2 L, KEQ-2 L and QM3SP2) were purchased from Tianchuang Powder Technology Co. (Changsha, China), Miqi Instrument Equipment Co. (Changsha, China), Ru Rui Technology Co. (Guangzhou, China) and Ru Rui Technology Co. (Guangzhou, China), respectively. The analytical balance (TG328A) was purchased from Balance instrument factory (Shanghai, China). The pumping equipment was purchased from Guohua Electric Co. (Shanghai, China). The vacuum drying oven was purchased from Anteing Electronic Instrument Factory (Shanghai, China). The muffle furnace (TDL-1800 A) was purchased from Keda Instrument Co (Nanyang, China).

### Production procedures

The flake graphite powder and sodium bicarbonate (NaHCO_3_) solid were mixture and loaded into the reaction tank containing steel balls according to the experimental design. The ball mill was started after adjust the suitable rotating speed. The GIC was obtained after the designed ball milling time, and then take out the mixture and put it into the muffle furnace. The temperature of the muffle furnace was adjusted from 150 to 300 °C to be suitable for the decomposition of NaHCO_3_. After the designed reaction time, the mixture was cooled, washed and dried, then the expandable GIC was obtained. Expanded graphite was obtained by high temperature expansion of expandable GIC at 950 ℃.

We also investigated the effects of different preparation parameters on the quality of expandable GIC products. Eight process factors (such as ball milling media, ball milling media size, ball milling time, ball mill speed, decomposition temperature, decomposition time, mass ratio of flake graphite to NaHCO_3_ and ball mill model) were designed and adjusted in the production process.

### Characterization

#### Morphological elucidation

Morphological information of samples was obtained by SU8020 Hitachi scanning electron microscopy (Tokyo, Japan).

#### Structural investigation

The molecular structure of the GIC product obtained was identified by X-ray diffraction. The samples were scanned and recorded using the X-ray diffractometer (Rigaku, Japan) with an X-ray generator from 15 to 60 of 2θ (Braff angle), using Cu/Ka irradiation at 55 mA and 60 kV. The structure information of product was obtained by FT-IR (IS50). The wave number range scanned was 4000−400 cm^-1^. After washed and dried, the powders and KBr were compacted into disks and analyzed.

### Determination of expansion volume

Expansion volume refers to the volume (unit mass) of GIC after expansion at a certain temperature, the unit is mL/g. Determine the expansion volume according to the national standard GB10698-89, the specific determine steps are as follows [10]: Firstly, a certain amount of the samples prepared according to the experimental method described in 2.2 was weighed by an analytical balance, and a quartz beaker (with scale) was put into the muffle furnace (adjustable temperature 100−1500 °C) that has been heated to 950 °C to preheat for 5 min, then add the sample into the quartz beaker, do not close the furnace door, and take it out immediately as long as it no longer expands. Read the average value of the highest and the lowest point on the top surface of the sample after expansion as the expanded volume of the sample (V). The expanded volume Z is calculated using the following formula:

1 $${Z}=\frac{{V}}{{m}}$$

V- Volume of sample after expansion (mL),

m- Mass of the sample (g).

Two parallel tests were performed for each measurement, and the allowable error of the results conformed the requirements of the GB10698-89 standard.

## Results and discussion

### Optimization of process parameters

#### Effect of ball milling time

**Fig. 1:**
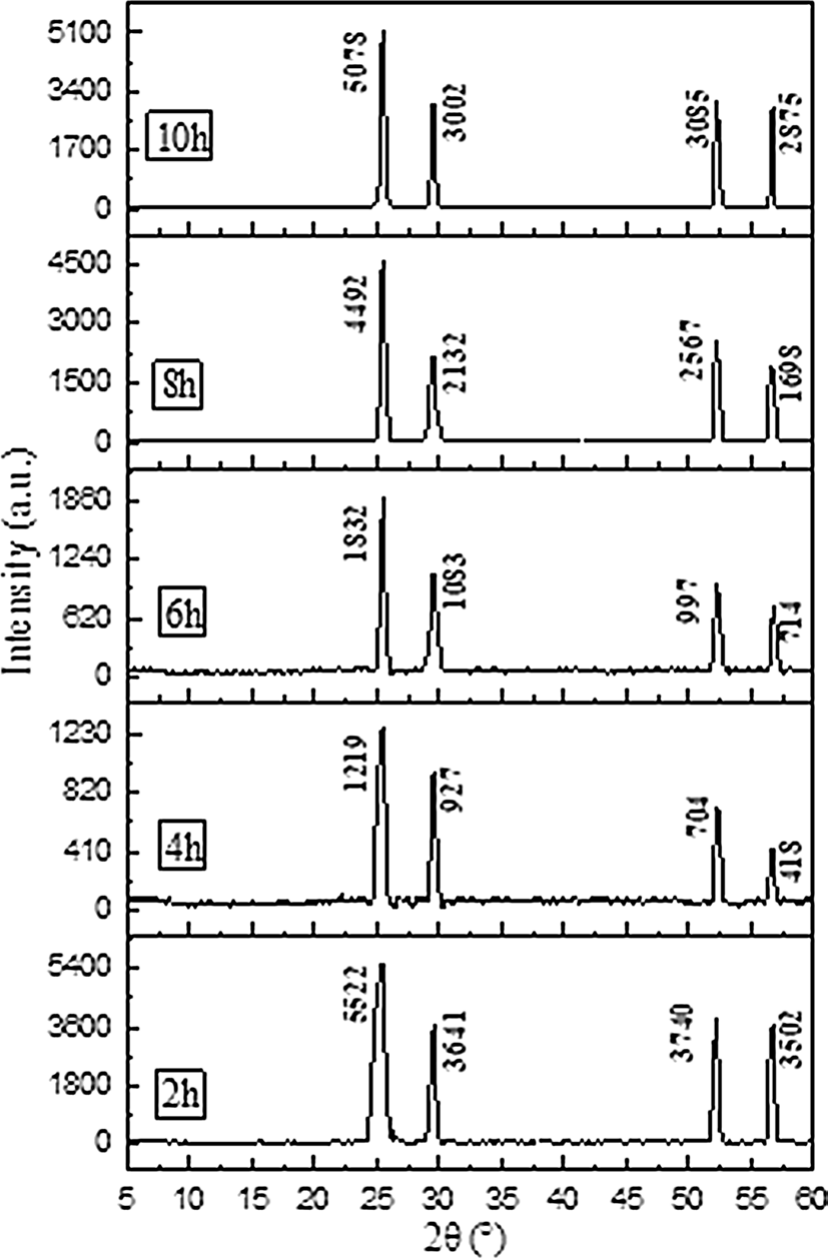
XRD patterns of GIC products after thermal treatment obtained by different ball milling times

**Fig. 2:**
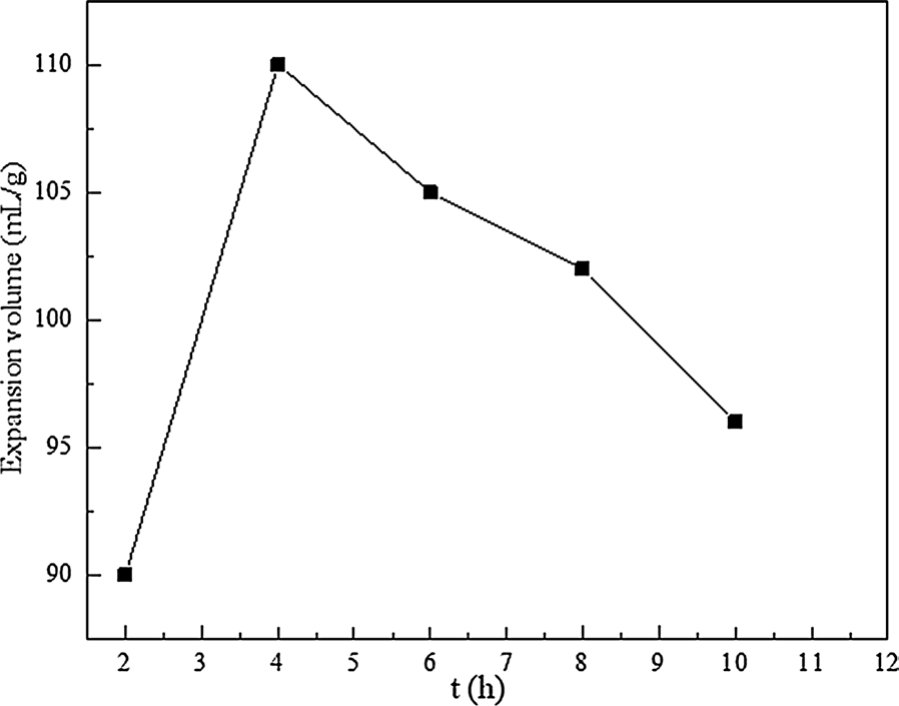
Expansion volumes of GIC products after thermal treatment obtained by different ball milling times

Figure 1 shows the XRD patterns of GIC products obtained from different ball milling times (2-10 h), and other experimental conditions were set as follows: ball mill speed was 500 r/min, the decomposition temperature was 150 ℃, the decomposition time was 2 h, the mass ratio of flake graphite to $\text{NaHC}{\text{O}}_{\text{3}}$ was 1:1. It can be seen from Fig. 1 that the samples prepared under different ball milling times all have the characteristic absorption peaks of GIC. Figure 1 shows when the time was extended, the intensity of the characteristic peak of GIC was decreased firstly and then increased, and reached the minimum at 4 h. According to literature reports, in the XRD analysis of GIC, the weaker intensity of the characteristic splitting peak and the larger peak width indicated the better intercalation effect. Generally speaking, ball milling is more sufficient as the ball milling time increases, and the mixing of graphite and intercalation agent are more uniform under the action of mechanical force, which leads to the decreased of the characteristic absorption peak. However, when the ball milling time is too long, the restacking of graphite is more pronounced, which is not conducive to the preparation of GIC. This resulted the increased of the intensity of the characteristic peak. Thus, the XRD characterization results shown that the intercalation effect was the best when ball milled for 4 h. Studies have shown that expandability is one of the important indicators of GIC products in practical applications. Therefore, the thermal expansion performance of GIC products obtained under different ball milling time has also been studied. And in the next single factor experiments, the expansion volume was used as the index to optimized the production parameters. Figure 2 shows the expansion volumes of GIC products obtained under different ball milling times. It can be seen that the expansion volume of GIC was increased firstly and then decreased when the ball milling time was extended, and reached the maximum at 4 h. Thus, the appropriate ball milling time was 4 h, which was adopted by the subsequent experiments run.

#### Effect of decomposition time

**Fig. 3:**
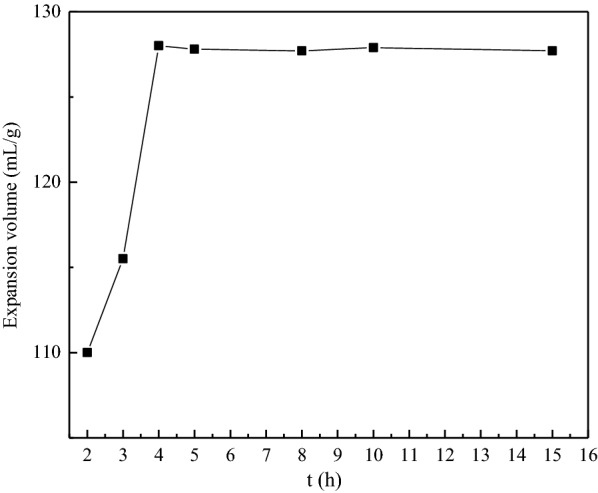
Expansion volumes of GIC products after thermal treatment obtained from different decomposition times

The effects of decomposition times on the production process were set as follows: ball milling time was 4 h, ball mill speed was 500 r/min, the decomposition temperature was 150 ℃, the mass ratio of flake graphite to NaHCO_3_ as 1:1 and decomposition times ranging between 1 and 15 h. Figure 3 shows the expansion volumes of GIC products obtained under different decomposition times. It shows when the decomposition time was extended, the expansion volume of GIC was increased firstly and then basically unchanged, and reached the maximum at 4 h. At the beginning, more carbon dioxide was produced by the decomposition of the intercalant NaHCO_3_ with the increase of the decomposition time, which can effectively increase the distance between the graphite flakes, lead to the expansion volume of GIC increased. However, when the decomposition time was prolonged, the intercalant NaHCO_3_ decomposed completed, and the decomposition product Na_2_CO_3_ cannot continue to decomposed (the decomposition temperature of Na_2_CO_3_ is above 850 ℃). This leads to the basically unchanged of the expansion effect. As it can be inferred from the results, 4 h decomposition time was found to be suitable for the investigation.

#### Effect of decomposition temperature

**Fig. 4:**
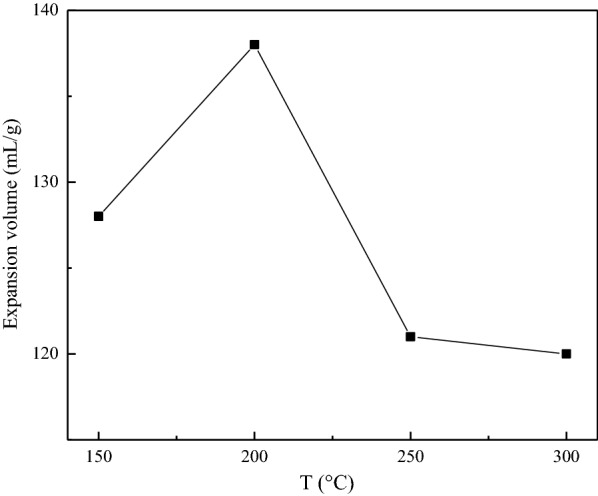
Expansion volumes of GIC products after thermal treatment obtained from different decomposition temperatures

The decomposition temperature is a key parameter, it directly affects the generated rate of gas obtained by the intercalation agent decomposed, which further affects the expansion effect [11]. Figure 4 shows the effect of the decomposition temperature on the production of GIC at the ball mill speed was 500 r/min, the mass ratio of flake graphite to NaHCO_3_ was 1:1 and the decomposition temperature was changed from 150−300 ℃. Figure 4 shows the expansion volumes of GIC products obtained under different decomposition temperature was increased firstly and then decreased, and reached the maximum at 200 ℃. This is because higher decomposition temperature resulted in better decomposition effect, which leads to an increase in the expansion volume of GIC. NaHCO_3_ solid starts to decompose at 50 ℃, and decomposes completely when the temperature reaches about 200 ℃. Therefore, when the decomposition temperature is too high, the decomposition rate is too fast, resulting in the carbon dioxide being lost without increasing the distance between the graphite layers, and the expansion effect is not good. From the results, a suitable decomposition temperature is 200 ℃.

#### Effect of ball mill speed

**Fig. 5:**
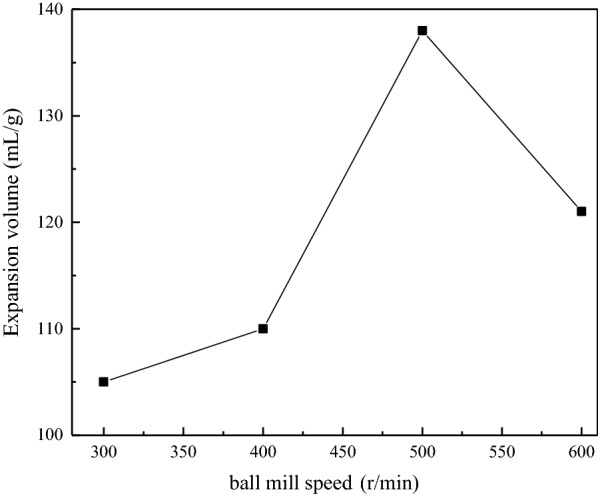
Expansion volumes of GIC products after thermal treatment obtained from different ball mill speeds

The effect of the ball mill speed on the preparation of GIC were performed from 300 r/min to 600 r/min, and the mass ratio of flake graphite to NaHCO_3_ was 1:1. Figure 5 shows the expansion volumes of GIC products obtained under different ball mill speed was increased firstly and then decreased, and reached the maximum at 500 r/min. This is because the more uniform mixing of graphite and intercalant under the influence of mechanical force when a higher ball mill speed is used. Meanwhile, the more restack of the graphene and uneven dispersion when the ball mill speed is too fast, results in the decline of the expansion volume of GIC. Therefore, it is reasonable to expect that a suitable ball mill speed may exist. In order to ensure the better preparation process, 500 r/min was selected as the optimal ball mill speed.

#### Effect of mass ratio of graphite to intercalant

**Fig. 6:**
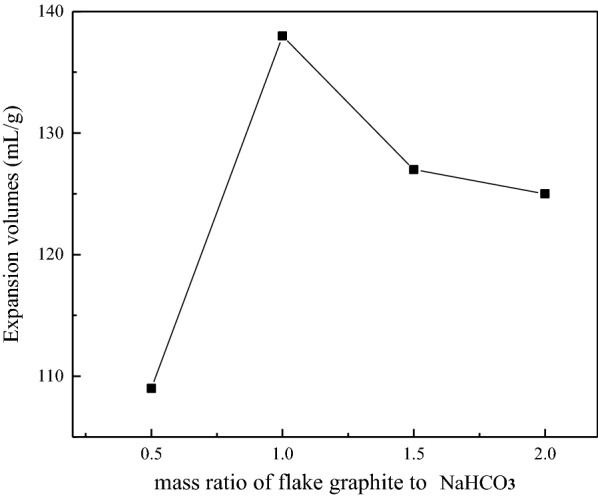
Expansion volumes of GIC products after thermal treatment obtained from different mass ratio of flake graphite to NaHCO_3_

Figure 6 shows the expansion volumes of GIC products obtained under different mass ratio of flake graphite to NaHCO_3_ was increased firstly and then decreased, and reached the maximum at 1:1. The mass ratio of flake graphite to NaHCO_3_ was adjusted from 1:0.5 to 1:2. This is because as the amount of NaHCO_3_ increases, it is beneficial to produce more carbon dioxide during decomposition and increase the distance between graphite layers, which leads to an increase of the expansion volume of GIC. But when the amount of NaHCO_3_ is too large, under the action of mechanical force, except for a small part mixed with graphite, most of the NaHCO_3_ is wrapped outside the graphite, and decomposes rapidly during thermal decomposition, resulting in poor intercalation effect, which leads to decrease in the expansion volume of GIC. From the results, a suitable mass ratio of flake graphite to NaHCO_3_ is 1:1.

#### Effect of ball milling media

The ball milling media have an impact on the production process, because different ball milling media have different squeezing force, impact force, shear force and internal sliding of the ball milling media on the ball milling process. Under the above process parameters, different ball milling media were used for the experiment. The specific experiment were as follows: zirconia ceramic beads, stainless-steel beads and cemented carbide beads were used as ball milling media (the diameter is 8 mm, and the number of ball milling media is 10). Table 1 shows the expansion volumes of GIC products obtained under different ball milling media. It can be seen that the expansion volume of the GIC obtained by zirconia ceramic beads as the ball milling medium was the smallest. This is due to the small specific gravity of the ceramic beads themselves, and the impact force, extrusion force and shear force on the ball milling material were small, and the ball milling efficiency was low, resulted in uneven mixing of graphite and NaHCO_3_. The ball milling effect of stainless-steel beads and cemented carbide beads were relatively good. This is because their own specific gravity was relatively large, and the kinetic energy generated by the drive of the ball mill was large, and the extrusion force, impact force and shear force of the ball mill material were larger. In addition, since the internal sliding of stainless-steel beads is greater than that of cemented carbide beads, which leads to the better grinding effect. Therefore, stainless-steel beads were used as the ball milling media.

**Table 1:** Expansion volumes of GIC products after thermal treatment obtained under different ball milling medias

Ball milling media	Zirconia ceramic beads	Stainless-steel beads	Cemented carbide beads
Expansion volume(mL/g)	98	138	129

#### Effect of ball mill media size

**Fig. 7:**
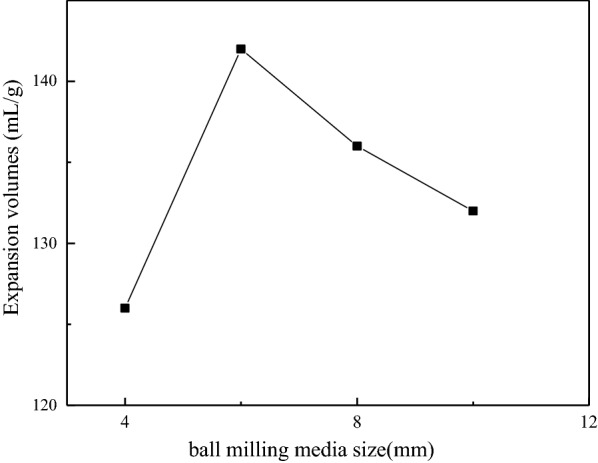
Expansion volumes of GIC products after thermal treatment obtained from different ball milling media sizes ball milling media sizes

The size of the ball milling media directly affects the grinding effect through the impact force, extrusion force and grinding effect on the material during the ball milling process. In order to ensure the same quality of the ball mill media loaded into the ball mill, stainless steel beads with diameters of 4 mm (0.26 g/piece), 6 mm (0.89 g/piece), 8 mm (2.1 g/piece) and 10 mm (4.16 g/piece) were used 80, 24, 10 and 5 for the experiment, respectively. Figure 7 shows the expansion volumes of GIC products obtained under different ball milling media size. The number of stainless-steel beads with a small diameter was large, and the striking force of each steel ball was small, but the number of strikes was large, and the grinding area was large. The number of stainless-steel beads with a large diameter was small, and the striking force of each steel ball was large, but the number of strikes was small, and the grinding area was small. Therefore, a good grinding effect can be achieved by choosing a suitable size of the ball milling media. It can be seen from Fig. 7 that when the diameter of the stainless-steel beads was 6 mm, the expansion volume of the GIC was the largest. This is because this size of the ball milling media, not only ensured the sufficient impact force, but also has more hit times and strong grinding effect. Hence, the diameter of 6 mm was selected.

#### Effect of ball mill model

In order to study the influence of different ball mill models on the preparation of GIC, experiments were carried out in four different models of ball mills according to the above optimal conditions. The experimental results were shown in Table 2. The ball mill manufacturers and models shown in the table are 1# (Changsha Tianchuang Powder Technology Co., Ltd. QM-3SP04), 2# (Changsha Miqi Instrument Equipment Co., Ltd. YXQM-2 L), 3# (Guangzhou Rurui Technology Co., Ltd. KEQ-2 L), and 4# (Guangzhou Rurui Technology Co., Ltd. QM3SP2), respectively. It can be seen from Table 2 that the expansion volumes of GIC prepared by using different types of ball mills under the same experimental conditions were basically the same. It can be seen that the production of GIC combined with mechanical force and chemical method described in this paper is stable and does no affected by the ball mill models.

**Table 2:** Expansion volumes of GIC products after thermal treatment obtained under different ball mill models

Ball milling model	1#	2#	3#	4#
Expansion volume(mL/g)	142	141	140	141

In summary, the optimal production conditions of GIC were as follows: the ball milling media was stainless-steel beads, the size of the ball milling media was 6 mm, ball mill time was 4 h, ball mill speed was 500 r/min, the decomposition temperature was 200 ℃, the decomposition time was 4 h, and the mass ratio of flake graphite to NaHCO_3_ was 1:1

At this condition, the expansion volume of GIC was 142 mL/g

### Mechanism discussion

#### Scanning electron microscope (SEM) analysis

**Fig. 8:**
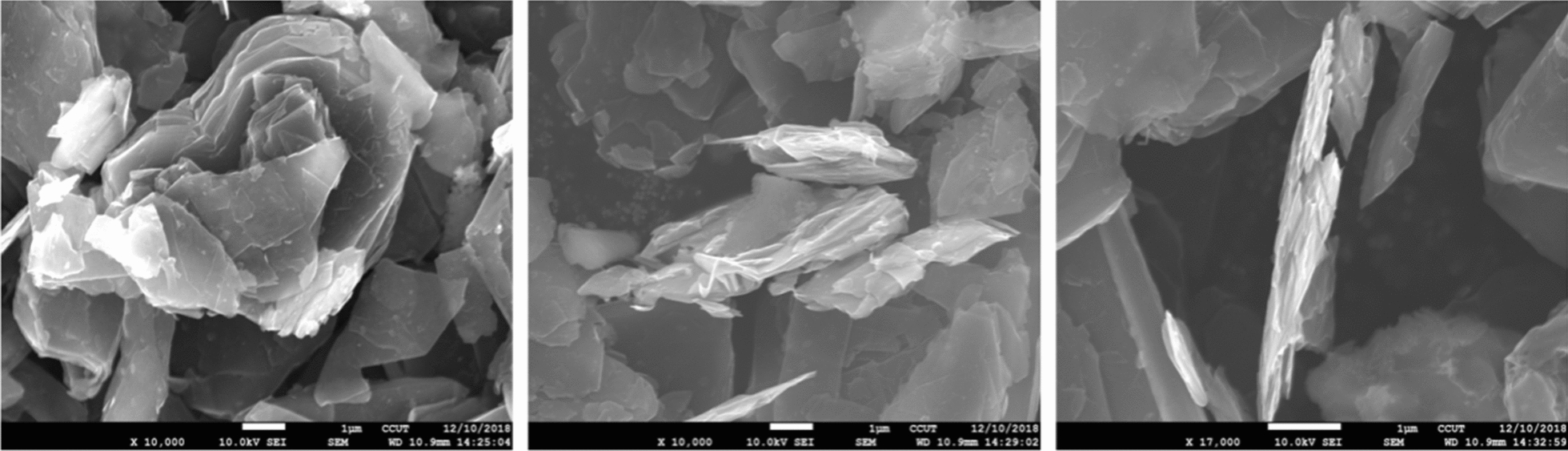
SEM images of GIC sample produced at the optimum conditions

Scanning electron microscope was used to observe the morphological of the GIC products obtained at optimum production conditions. It can be seen from Fig. 8 that GIC was composed of many bonded and superimposed graphite flakes [12]. The densely arranged graphite flakes were divided into graphite flakes with a thickness of several hundred nanometers, and there were obvious signs of bulging and swelling. This is due to the changes in the carbon layer structure caused by the intercalation agent entered between the graphite layers. Due to the intercalation effect, many honeycomb-like fine pores appear between the graphite flakes, and the pores were fusiform. The layered structure still exists, but fractures and voids appear between the lamellae. This is because the van der Waals force between the layers was destroyed and the distance between the lamellae increased significantly under the effect of the intercalation.

#### X-ray diffraction (XRD) analysis

**Fig. 9:**
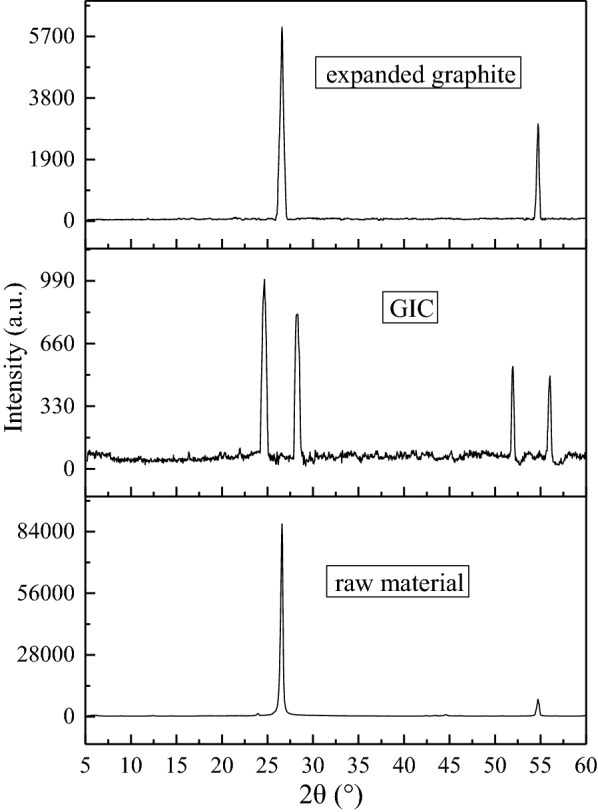
XRD patterns of different samples

In order to study the crystal structure change of the product before and after intercalation, the XRD pattern of the samples were measured. Figure 9 shows the XRD patterns of graphite raw materials, GIC and expanded graphite, respectively. The expandable GIC product was obtained at optimum production conditions. Expanded graphite was obtained by high temperature expansion of GIC at 950 ℃. It can be seen from Fig. 9 that natural flake graphite has two characteristic sharp peaks at.

2θ = 26.60° and 2θ = 54.76° [13], and the diffraction peak intensity is large, which is due to the regular arrangement of internal particles and high crystallinity. The intensity of the diffraction peaks of GIC were greatly weakened, and the peaks width were broadened. The d_002_ diffraction peak (2θ = 26.60°) was split into two diffraction peaks 2θ = 24.68° and 2θ = 28.32° and the d_004_ diffraction peak (2θ = 54.76°) was split into two diffraction peaks 2θ = 51.98° and 2θ = 56.02°. This is because after the flake graphite was intercalated, the distance between the graphite flakes increased and the crystal structure was damaged, which resulted the split and left shift of the diffraction angle and the weak of the diffraction intensity. It was known that the interlayer spacing can be calculated according to the Bragg Eq. 2dsinθ = nλ [14, 15]. It was known that λ = 1.54 nm under the test conditions, the interlayer spacing of GIC were 0.366 and 1.75 nm calculated by substituting 2θ = 24.68° and 2θ = 51.98° in Fig. 9 into the Bragg equation respectively, which is larger than the interlayer spacing of flake graphite by 0.335 and 1.67 nm respectively. This is due to the destruction of the structure of the graphite along the C axis direction, which indicated that the NaHCO_3_ intercalation agent has entered the interlayer of the graphite, and increased the interlayer distance of the layered graphite. The above results indicated that the intercalation agent has entered between the graphite layers, and GIC was prepared. The interlayer structure of the expanded graphite obtained after the expansion of GIC was partially destroyed due to the effect of the intercalator. The remaining undestroyed graphite crystallites still retain the original graphite structure, so the characteristic diffraction peaks of expanded graphite were basically the same as that of flake graphite. The diffraction peak intensity of expanded graphite was significantly weakened and the peak shape was sharp compared with the flake graphite, which indicated that the crystallites in the expanded graphite were further reduced, but still have graphite crystallites.

#### Fourier transform infrared spectra (FT-IR) analysis

**Fig. 10:**
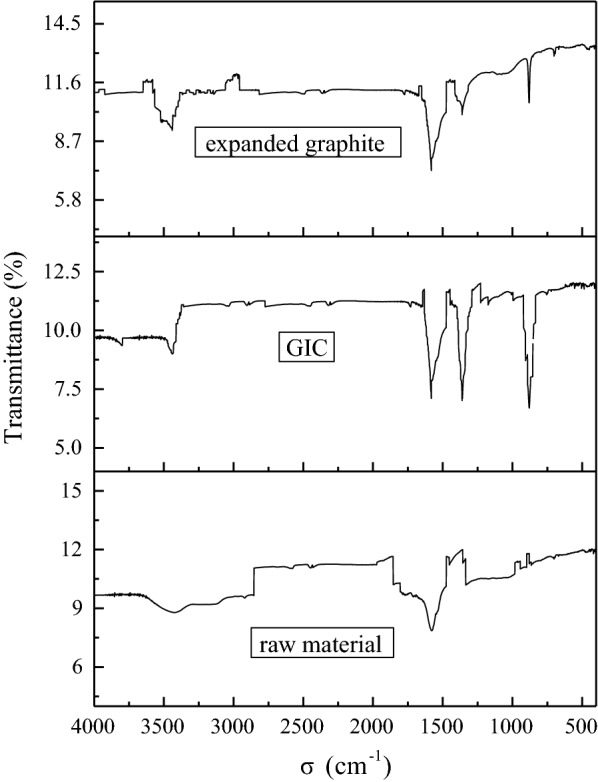
FT-IR patterns of different samples

As a relatively easy method, FT-IR spectroscopy has been widely used in GIC research, from which the direct structural information and changes can be obtained during various chemical treatments. Figure 10 shows the FT-IR patterns of graphite raw materials, expandable GIC obtained at optimum production conditions and expanded graphite, respectively. It can be seen that the three samples all have characteristic absorption peaks at 1582 cm^-1^ and 3428 cm^-1^. The absorption peak of 1582 cm^-1^ was belonged to the sp^2^ structure of graphite crystal C = C stretching vibration peak [16], indicated that the internal structure of the GIC and the expanded graphite layer has not changed, and the preparation method does not change the carbon-based structure. The absorption peak at 3428 cm^-1^ attributed to OH stretching vibration peaks, which is the trace moisture contained in the sample itself or KBr when pressed. In the infrared spectrum of GIC, there are strong characteristic peaks in 880 cm^-1^ and 1360 cm^-1^. The peak at 880 cm^-1^ was caused by the carbonate internal stretching vibration mode, and 1360 cm^-1^ was the absorption peak of carbonate internal stretching vibration mode [17]. The above results indicated that the presence of carbonate in GIC. It can be seen from the infrared spectrum of expanded graphite that the characteristic peak of carbonate was significantly weakened, indicated that the acid radical ions have decomposed to gas and escaped, but there was still a small amount of residue. These results further indicated that the preparation method does not change the carbon-based structure, and the NaHCO_3_ intercalation agent has entered the interlayer of the graphite, which increased the interlayer distance of the layered graphite.

#### X-ray photoelectron spectrometer (XPS)

**Fig. 11:**
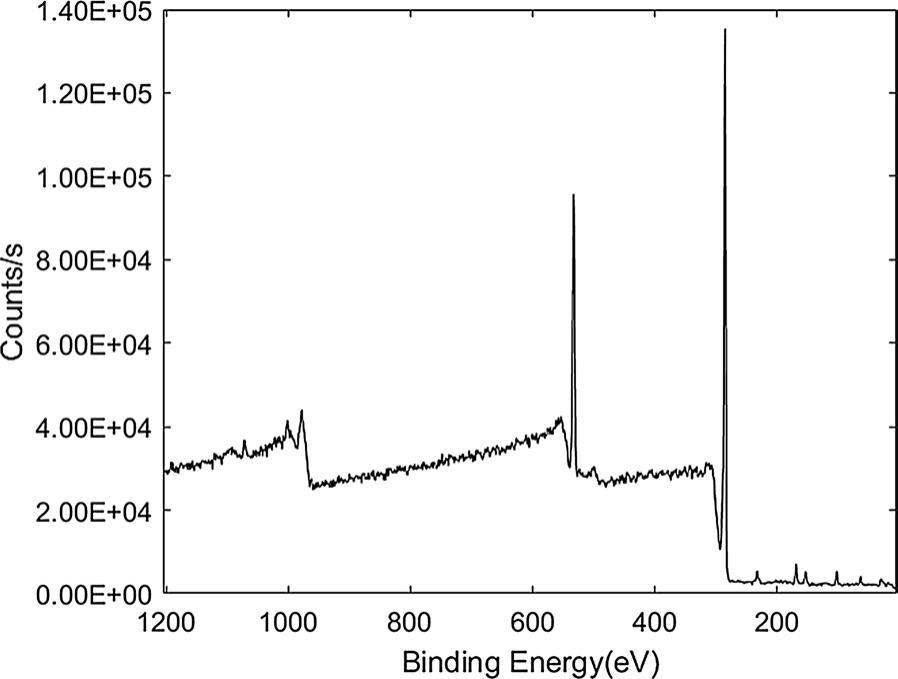
XPS patterns of GIC sample produced at the optimum conditions

To further analyse the elements of the GIC product, we used the XPS test. The experimental results are shown in the Fig. 11. From Fig. 11, The Bindding Energy at 282.55 ev and 530.33 ev were GIC’s characteristic peaks which attributed to the C1s and O1s. C1s is mainly due to the carbon structure of GIC, and O1s is mainly due to the intercalator. Further quantitative calculations found that the carbon element content of the GIC product was 88.98%, and the oxygen element content was 11.02%. The experimental results show that our preparation method produces good GIC products with no impurities. This result of XPS is in good agreement with those of FT-IR and XRD.

##### Production mechanism

**Fig. 12:**
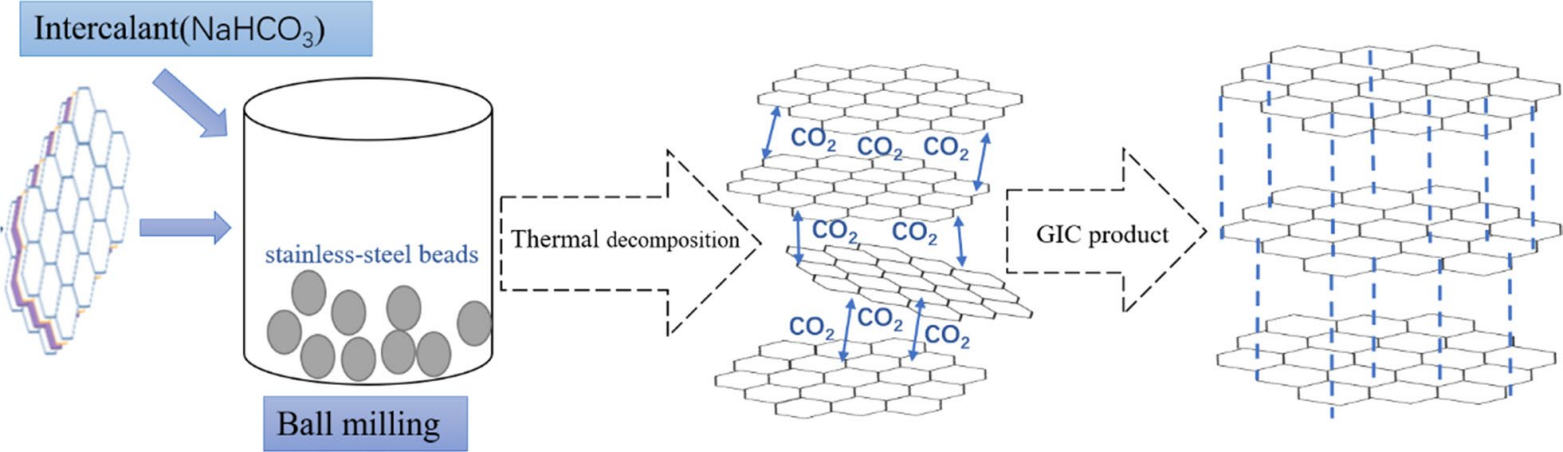
Schematic representation of the production mechanism

The schematic representation of the production mechanism can be seen as Fig. 12. The flake graphite is intercalated with NaHCO_3_ as an intercalant under the action of mechanical ball milling, and then NaHCO_3_ was decomposed at a suitable temperature under the protection of inert gas. The gas generated during the decomposed of NaHCO_3_ increases the interlayer spacing of the layered graphite, and the GIC product was obtained after washed and dried. Further research shown that the GIC prepared by this method has good thermal expansion properties. The method has the advantages of simple process, mild preparation conditions, no use of aggressive reagents, process stability, etc. Thus, it could be an alternative green and efficient method for GIC production in industry.

#### Process flow design of the preparation method

**Fig. 13:**
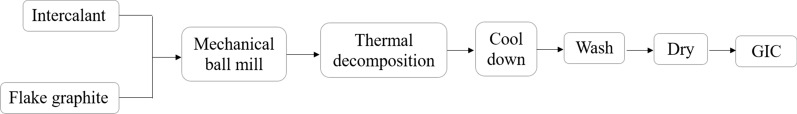
Process flow diagram of preparation method

According to the experimental results in this paper, we designed the process flow for GIC green production by the combined with mechanical force and chemical method. The specific process flow chart is shown in Fig. 13. After mixing the graphite and the intercalant in a certain ratio, perform mechanical ball milling at a set speed. After the set ball milling time was reached, the mixture was taken out, and then the mixture was thermally decomposed at a suitable temperature. After reached the decomposition time, expandable GIC can be obtained after cooled, washed and dried.

## Conclusions

This paper investigated a new method by the combination of mechanical force and chemical method to produce the GIC from graphite. The effect of production conditions on the thermal expansion performance of GIC products were investigated by single factor experiments, the optimal conditions were obtained as 6 mm diameter of the stainless-steel beads ball milling media, the ball mill speed 500 r/min for 4 h, the decomposition temperature 200 ℃ for 4 h, and the mass ratio of flake graphite to NaHCO_3_ was 1:1. Under optimized conditions, the expansion volume of GIC product was 142 mL/g. At the same time, the mechanism of preparation method was studied, and the preparation process was designed. The method has the advantages of simple process, products free of impurities, no use of aggressive reagents, process stable, etc. In general, the new method could be a green and potential method for GIC production in industry.

## Data Availability

All data generated or analysed during this study are included in this published article.
